# Intergenerational transfer of time and risk preferences

**DOI:** 10.1016/j.joep.2015.06.003

**Published:** 2015-08

**Authors:** Heather Brown, Marjon van der Pol

**Affiliations:** a Institute of Health and Society, Newcastle University, UK; b Health Economics Research Unit, University of Aberdeen, Scotland

**Keywords:** Intergenerational transfer, Risk preference, Time preferences, Generalised ordered probit, Australia

## Abstract

There is a growing interest in individual time and risk preferences. Little is known about how these preferences are formed. It is hypothesised that parents may transmit their preferences to their offspring. This paper examines the correlation in offspring and parental time and risk preferences using data from an annual household survey in Australia (the HILDA survey). Both time and risk preferences are examined and we explored whether the correlation in time and risk preferences varies across the distribution of preferences and across the across the four parent–child dyads (mother/daughter, mother/son, father/daughter, father/son). The results show that there is a significant relationship between parents and their young adult offspring risk and time preference measures. The correlation varies across the distribution of time preferences. The correlation was largest for longer planning horizons. Risk averse parents are more likely to have risk averse children. Except for the father/daughter dyad risk seeking parents are more likely to have risk seeking offspring. Some gender differences were found. The association in parental and offspring time preference was larger for mothers than fathers. Daughters are more likely to be influenced by their mother’s risk preferences, however, sons are equally influenced by both parents. The results of this study suggest that the transmission in preferences is more nuanced than previously thought and parental gender may be important.

## Highlights

• Explores correlation in offspring and parent time and risk preference using Australian panel data.
• We examine correlations across the preference distribution and by gender.
• Evidence of correlation in offspring risk and time preferences was found.
• Largest correlation in time preference was found for the longest planning horizons.
• Gender differences in the preference correlations were found.

## 1 Introduction

Time and risk preferences are key parameters in economic models determining consumption and savings over the lifecycle. They play an important role in an individual’s decision to invest in education, pensions, health, etc. Risk and time preference were originally thought of as fixed parameters. Empirical evidence suggests that time and risk preferences vary considerably between individuals (Barsky, Kimball, Juster, & Shapiro, 1997; Frederick, Loewenstein, & O’Donoghue, 2002). This has also been shown within the psychology literature with respect to related concepts such as time perspective, delay of gratification, risk taking propensity and attitudes to risk taking. These empirical findings suggest that the notion of risk and time preference as fixed parameters needs to be revised. This has led to increased interest by economists in how risk and time preferences are formed and how they evolve over the lifecycle.

Little is known about how preferences are formed. Individuals may be born with innate time and risk preferences and/or preferences may be learned. There is some evidence that preferences are determined by the genetic makeup of the individual. For example, Carpenter, Garcia, and Lum (2011) show that certain genotypes are associated with time and risk preferences. However, evidence shows that preferences vary over the lifecycle which suggests that preferences may be endogenous (Camerer & Loewenstein, 2004). Becker and Mulligan (1997) develop a model of endogenous time preferences to understand how parents may influence their offspring’s time preferences other than through genetics. In this model individuals can invest resources to make future consumption seem less remote. They do this because they are aware that heavy discounting of the future is undesirable. Resources include time and effort spend appreciating future consumption but also the purchase of precommitment devices such as Christmas Clubs. In this model parents can influence offspring time preference by investing resources in teaching their children to better plan for the future. This framework can be extended to risk preferences in that individuals can invest resources to become more risk averse. Parents can influence their offspring risk preferences by investing resources in teaching them to be more risk averse.

In this paper we are interested in the transmission of preference parameters from parents to their offspring. It is acknowledged that this may occur through both genetic inheritance and learning. There is limited empirical evidence of intergenerational transfer of time and risk preferences. Four previous studies have examined correlations in time preference between parents and their offspring all using relatively small sample sizes and using a range of different measures of time preference including future orientation, saving residuals and rate of time preference. Webley and Nyhus (2006) used data from three waves of the DNB household survey to examine whether parents and children’s’ future orientation is correlated (*n* = 308 children aged 16–21). Future orientation could be argued to be related to the concept of time preference. A significant correlation of 0.28 was found between father and children future orientation and 0.31 was found between mother and children’s future orientation. Knowles and Postlewaite (2005) investigated correlations in time preference (saving residuals) between parents and their children using data from the Panel Study of Income Dynamics (PSID) (*n*∼1300 aged 1–25). They find a significant correlation in saving residuals which ranges from 0.11 to 0.22 depending upon the model specification. Reynolds, Leraas, Collins, and Melanko (2009) estimated correlations in time preference rates between mothers and their offspring in a small sample (*n* = 30 children aged 12–13). They found a correlation of 0.29 in time preference rates but the correlation was not statistically significant. Kosse and Pfeiffer (2012) examined correlation between time preference rates in mothers and delay of gratification in pre-school children (*n* = 213 preschool children). A significant correlation was found in case of a short term (6-month) time preference rate (ranging from 5.2 percentage points to 6.8 percentage points depending upon the model specification) but longer term (12 months) time preference rate did not have a significant effect on child’s impatience.

A similar number of studies have examined correlations in risk preferences between parents and their offspring. Dohmen, Falk, Huffman, and Sunde (2012) investigated the intergenerational transmission of risk preferences measured using a general question regarding willingness to take risk from the German Socio-Economic Panel (*n* = 3595 children aged 17–54). The results show that risk preferences of parents and their children are significantly correlated (0.149 for mothers and 0.153 for fathers). Hryshko, Luengo-Prado, and Sorensen (2011) and Charles and Hurst (2003), using similar analyses and similar data (PSID), found that risk preferences, measured using a gamble with different levels of lifetime income, were correlated but at the more extreme end of the distribution only (*n* = 583 children). Hrysko et al. show that risk seeking parents are 13% less likely to have children that are risk averse. This result is very similar to that found in Charles and Hurst (2003), however they also find a significant correlation for risk aversion between parents and offspring ranging from 0.123 to 0.154 depending upon the controls included in the model. Allowing for measurement error, Kimball, Sahm, and Shapiro (2009), also using the PSID find significant correlations in risk preference of 0.23 between mothers and their children and 0.14 between fathers and their children. Arrondel (2013) is the only study that considered both risk and time preferences. Risk and time preferences were measured using a score based on a series of questions on risk taking behaviour (e.g. whether the individual gambles) and time preference (e.g. whether sacrifice today in order to live longer). Using French data, statistically significant raw correlations of approximately 0.25 for both measures (*n* = 440 children who were on average aged less than 34 and parents who were on average 59 years old). However, once controls were included in the analysis the correlations were no longer symmetric. The elasticity in parent and offspring risk preference is 0.277 and was 0.122 in parent and offspring time preference.

The empirical studies show a modest correlation in time and risk preferences between parents and their offspring. However, all studies apart from Webley and Nyhus (2006) are cross sectional analyses and several studies have small sample sizes. This paper investigates the correlation in parental and offspring time and risk preferences using data on a larger sample with proxies of time and risk preference across several waves. The availability of panel data allows us to control for unobserved heterogeneity, i.e. both unobserved time invariant genetic factors and unobserved time varying factors impacting on the correlation in parental preferences. The availability of a larger sample and the availability of data on all members of the household also allows us to examine the correlation in parental and offspring preferences across the four parent–child dyads (mother/daughter, mother/son, father/daughter, and father/son). Gender may have a powerful and persuasive relationship within the family. This includes distinctness in terms of the characteristics of the relationship such as the content and style of interactions. Research has shown that fathers are more likely to be involved with male offspring and mothers with female offspring (Harris & Morgan, 1991; Younis & Smollar, 1985) and that affective intensity is strongest across same gender lines (Steinberg, 1987). The extent of interaction and the intensity of the relationship is likely to have an influence on the transmission of preferences. It is therefore important to explore whether the correlation in time and risk preferences varies across the four parent–child dyads (mother/daughter, mother/son, father/daughter, and father/son). It is hypothesised that the transmission of preferences is strongest along the same gender lines. This is to our knowledge the first study to examine the correlation in time and risk preferences across the four parent–child dyads. We also explore whether the correlation varies across the distribution of preferences. For example, it may be the case that risk preferences are correlated at the extreme ends of the preference distribution (very risk averse or very risk seeking) only. This would mean that whilst the overall correlation is small parental preferences may have a substantial effect for some of the offspring. Charles and Hurst (2003) find a correlation in risk preferences at the tail end of the spectrum only (very low and very high risk tolerance) using a series of linear probability models. We explore this in both time and risk preferences using a more efficient estimation technique, namely (generalised) ordered probit regression. Exploring how time and risk preferences are correlated across the spectrum of these variables and if the parent/children correlation is the same across the four parent–child dyads is important for understanding the mechanisms behind the transmission in preferences.

## 2 Data

The data used in the analysis comes from waves 2, 3, 4, 6, 8, 10, and 11^1^ of the Household Income Labour Dynamics of Australia (HILDA).^2^ The HILDA is an annual household based panel survey which began in 2001. The panel members are followed over time and each household member over the age of 15 is interviewed. This is an advantage compared to the Panel Study of Income Dynamics (PSID) used by Charles and Hurst (2003) where the main questionnaire is limited to the household head. The survey is unique among household surveys in collecting multiple year information on financial, demographic, and health information for a general population.

The analysis is restricted to young adults between the ages of 16–25 and their parents. Time and risk preferences were elicited in respondents aged 16 and over only. The 16–25 range was of main interest as this is an important age range for making education and employment decisions that will affect the lifetime income stream. As time and risk preference are key factors in influencing these decisions it is important to understand the relationship with parental preferences at this pivotal age. Whilst it would have been interesting to explore whether the correlation is different for older age groups when it could be argued that parents have less of an influence, the sample size of the older age group limited the scope for analysis (*n* = 100 for males and *n* = 54 for females in the 25–61 in age range). The majority of the sample (95%) resides with their parent(s) as either dependent students or non-dependent children. Respondents without mothers in the survey are not included in our estimation sample. Respondents with fathers in the survey are matched to their father’s survey responses. Approximately 75% of the total sample have both mother and father information. There are observations for *n* = 2757 males, *n* = 2555 females, *n* = 2965 mothers and *n* = 2338 fathers.

### 2.1 Dependent variables

#### 2.1.1 Time preference

Within the economics literature time preferences are usually elicited using hypothetical trade-offs between different outcomes over time with real incentives. However, given the relatively high cost of collecting these data these measures are usually not included in household surveys and empirical studies therefore have to rely on proxies. A commonly used proxy for time preference is financial planning horizon. This question is asked to respondents in the self-completion questionnaire:

“In planning your savings and spending which of the following time periods is most important to you?”

The respondent has the option of choosing: (1) next week; (2) next few months; (3) next year; (4) next two to four years; (5) next five to ten years; and (6) more than ten years ahead.

It is hypothesised that individuals with shorter planning horizons have higher time preference rates than individuals with longer planning horizons. Planning horizon has been used in several studies as a proxy for time preference (see for example Picone, Sloan, & Taylor, 2004, Khwaja, Sloan, Salm et al., 2006, Samwick, 1998). Adams and Nettle (2009) show that planning horizon and discount rate, measured using hypothetical trade-offs over time, are correlated, −0.19, (*p* value < 0.001). While it is plausible that individuals with a higher time preference rate have a shorter planning horizon, socio-economic status and life expectancy are also likely to be associated with length of planning horizon. The data available in the HILDA allows us to control for socio-economic status and age.

We created an ordered categorical variable based on the six choices where the base category is a planning horizon of next week and each subsequent category progresses to a longer planning horizon. An individual with a low rate of time preference should have a long planning horizon and vice versa. The distribution of the time preference for males and females and their parents is presented in Table 1. A higher percentage of males report a planning horizon of the next week and a higher percentage of females report a planning horizon of a year suggesting that males in this age group may exhibit a higher rate of time preference. The majority of mothers report a higher rate of time preference (planning for the next week or few months). Father’s reported time preference is more spread out throughout the categories than mother and offspring time preference. Approximately equal percentages of father’s report planning horizons of the next week, next year, and next 5–10 years. A higher percentage of fathers appear to exhibit a lower rate of time preference than mothers and offspring. This may reflect the traditional role of fathers as the primary income earners in charge of the family finances. Our sample is limited to fathers who remain in a stable relationship with the offspring mother which may be different to fathers that separate from their children’s mother and the male population in general.

#### 2.1.2 Risk preference

The Survey of Consumer Finances (SCF) risk-tolerance measure is used as a proxy for risk preferences. This question is asked to respondents in the self-completion questionnaire:

“Which of the following statements comes closest to describing the amount of financial risk that you are willing to take with your spare cash?” That is cash used for savings and investment.

The options given to the respondent are: (1) I take substantial financial risk expecting to earn substantial returns; (2) I take above average financial risks expecting to earn above average returns; (3) I take average financial risks expecting to earn average returns; (4) I am not willing to take any financial risks; (5) I never have any spare cash. If the respondent reports that they never have any spare cash, they are asked to imagine what they would do if they had any spare cash available for investment and savings using the first four options from above.

This measure is widely used as a proxy for risk preferences. Grable and Lytton (2001) show in their review of the literature that this measure is stable over time and is correlated with investments in risky assets. Hanna and Lindamood (2004) showed that the risk tolerance measure is correlated with risk preferences measured using lotteries with different retirement income as outcomes. Lotteries are the most popular method of eliciting risk preferences within the field of economics. While it is plausible that individuals who are not willing to take risks with spare cash as risk averse socio-economic status and life expectancy are also likely to be associated with willingness to take risk. The data available in the HILDA allows us to control for socio-economic status and age.

The response categories can be interpreted as going in ascending order from risk averse, to risk neutral, to risk seeking. An ordered categorical variable was therefore created which equals zero if the respondent is not willing to take any financial risks (risk averse), is equal to one if the respondent will take average risks for average returns (risk neutral), and is equal to two if the respondent will take above average or substantial risks (risk seeking). Respondents that report never having any spare cash are coded into the above categories given their hypothetical answers to what they would do if they did have spare cash. The distribution of the categorical risk variable for young adults and their parents are presented in Table 1. A higher percentage of females are not willing to take financial risks compared to males. Additionally, a higher percentage of males report risk seeking behaviour compared with females. The majority of mothers are risk averse whilst the majority of fathers are risk neutral. However, a higher percentage of fathers declare themselves to be risk seeking than mothers.

The time preference proxy is observed over 8 years in 6 waves and the risk preference proxy over 4 years in 4 waves and it can therefore be explored how stable the measures are over time. Under the standard economic assumption of stable preferences we would not expect any variation in the preference proxies over the waves. However, there may be confounding variables such as income that change over time or preferences may change over the lifecycle (Becker & Mulligan, 1997). However, given the relatively short time frame we expect a certain level of persistence unless there is considerable measurement error. We calculate transition matrices for the time and risk preference measure for young adults and their parents. The young adult matrices are shown in Tables 1A and 1B.^3^ Tables 1A and 1B show that there is some persistence in preferences between periods *t* and *t−*1 which we can see by observing the higher probabilities on or close to the diagonals and the lower probabilities away from the diagonals. There are some exceptions such as 25% of males that report a planning horizon of 10 or more years this year had reported a planning horizon of a week or a few months the previous year. We further explore the within individual evolution in preferences for the respondents who we have observations for over the period when they are between the ages of 16–25. Time preferences within individuals are fairly consistent within this age range. There is some movement to becoming one category more future orientated (risk averse) and one category more present orientated (risk seeking) which is consistent with the transition matrix for the full sample. For the main econometric analysis, because we follow the same individuals over time, we can control for observable and unobservable factors that may impact on a change in risk and time preference over time. Our flexible model specification means that the explanatory variables do not need to have a homogenous effect on the different time and risk preference categories. Life events such as finishing one’s education, getting a first job, or moving out of the family home may have a temporary impact on preferences which we are observing in the raw data.

### 2.2 Explanatory variables

The proxies for time and risk preferences used in this paper both relate to financial behaviours, financial planning horizon for savings and spending and risk taking with spare cash. These measures have been shown to be correlated with time and risk preferences measured using more standard ways of eliciting time and risk preferences within the economics literature. However, there are also likely to be several confounding variables such as economic, demographic factors, and parental characteristics. It is therefore important to control for these variables in the regression models. In addition, the 16–25 year old age is a large age range covering a formative period in an individual’s development and life experience. In the econometric analysis we include age as a covariate which will control for any difference in life experience that may impact on preferences. A full list and explanation of the explanatory variables included in the analysis can be found in Table 2. Health behaviours (frequent physical activity, frequent binge drinking, and smoking variable) are also included to validate the time and risk preference proxies, that is, it is examined whether are they associated with real behaviour. The models are also estimated without the health behaviour variables because of potential endogeneity problems with these variables.

### 2.3 Descriptive statistics

The descriptive statistics for the estimation sample are presented in Table 1. 61% of females and 53% of males are full time students and 68% of males engage in physical activity at least 3 times per week compared to 50% of females; two activities which are likely to be associated with being future orientated and engaging in less risky behaviour. 13% of females report currently smoking whereas 19% of males report being current smokers an activity associated with present orientated and risky behaviour. Looking at parent characteristics, approximately 44% of mothers and 24% of fathers have a higher degree, 22% of parents are smokers, 50% of fathers and 44% of mothers participate in physical activity at least three times a week all activities which are likely to be associated with time and risk preferences.

## 3 Econometric model

Our two dependent variables, time and risk preference are classified as ordered categorical variables. Ordered probit models rather than linear regression are used to allow us to examine whether the correlation in preferences between parents and their offspring varies across the preference distribution. To adequately capture heterogeneity in both the error term and the dependent variables we estimate random effects generalised ordered probit models (Pfarr, Schneider, & Schmid, 2010).

The base model is a random effects ordered probit model that assumes homogenous cut-points between the categories of the dependent variable. The main estimation model is a generalised random effects ordered probit model. Let the time preference proxy, H be a discrete ordered variable taking the values of 0 if the young adult reports a planning horizon of the next week, 1 if planning horizon is the next month, 2 if planning horizon is the next year, 3 if planning horizon is the next two to four years, 4 if planning horizon is the next five to ten years and 5 if planning horizon is for more than ten years.

Let the risk preference proxy R be a discrete ordered variable taking the value of 0 if the young adult reports being risk averse, 1 if risk neutral, and 2 if risk seeking.

The model can be expressed for time preference and risk preference respectively as:

(1) $$H_{\mathrm{it}}=j\;\text{if}\;\mu_{j-1}<H_{\mathrm{it}}^{*}\leq \mu_{j}\text{,}\;\;j=1\text{,}\ldots \text{,}5\text{.}$$

(2) $$R_{\mathrm{it}}=k\;\text{if}\;\mu_{k-1}<R_{\mathrm{it}}^{*}\leq \mu_{k}\text{,}\;\;k=1\text{,}2$$

where the latent variables $H_{\mathrm{it}}^{*}$ and $R_{\mathrm{it}}^{*}$ are assumed to be linear functions of time varying economic factors represented by the vector $X_{\mathrm{it}}$, time varying and time constant demographic factors captured in the vector $Q_{\mathrm{it}}\text{,}$ and time varying and time constant parental characteristics included in the vector $P_{\mathrm{it}}$, plus a random error term, $\varepsilon_{\mathrm{it}}$ which is comprised of an idiosyncratic error term $\mu_{\mathrm{it}}$ and individual effects $\alpha_{i}$.

(3) $$H_{\mathrm{it}}^{*}=\beta X_{\mathrm{it}}+\xi Q_{\mathrm{it}}+\psi P_{\mathrm{it}}+\varepsilon_{\mathrm{it}}$$

(4) $$R_{\mathrm{it}}^{*}=\beta X_{\mathrm{it}}+\xi Q_{\mathrm{it}}+\psi P_{\mathrm{it}}+\varepsilon_{\mathrm{it}}$$

The observed and coded time preference proxy, $H_{\mathrm{it}}$ and risk preference proxy $R_{\mathrm{it}}$ are determined from the models as follows:

(5) $$H_{\mathrm{it}}=\left\{\begin{array}{c} 0\;\text{if}\;-\infty \leq H_{\mathrm{it}}^{*}\leq \mu_{1}\;(\mathrm{next}\;\mathrm{week})\\ 1\;\text{if}\;\mu_{1}<H_{\mathrm{it}}^{*}\leq \mu_{2}\;(\mathrm{month})\\ 2\;\text{if}\;\mu_{2}<H_{\mathrm{it}}^{*}\leq \mu_{3}\;(\mathrm{year})\\ 3\;\text{if}\;\mu_{3}<H_{\mathrm{it}}^{*}\leq \mu_{4}\;(2\;\text{to}\;4\;\mathrm{years})\\ 4\;\text{if}\;\mu_{4}<H_{\mathrm{it}}^{*}\leq \mu_{5}\;(5\;\text{to}\;10\;\mathrm{years})\\ 5\;\text{if}\;\mu_{5}<H_{\mathrm{it}}^{*}\leq \infty \;(10\;\mathrm{years}+) \end{array}\right)$$

(6) $$R_{\mathrm{it}}=\left\{\begin{array}{c} 0\;\text{if}\;-\infty <R_{\mathrm{it}}^{*}\leq \mu_{1}\;(\mathrm{risk}\;\mathrm{averse})\\ 1\;\text{if}\;\mu_{1}<R_{\mathrm{it}}^{*}\leq \mu_{2}\;(\mathrm{risk}\;\mathrm{neutral})\\ 2\;\text{if}\;\mu_{2}<R_{\mathrm{it}}^{*}\leq \mu_{3}\;(\mathrm{risk}\;\mathrm{seeking}) \end{array}\right)$$

where $\mu_{j}$ and $\mu_{k}$ represent the cut-off points to be estimated along with the coefficient vectors β,ξ, and ψ in Eqs. (3) and (4) for time preference and risk preference respectively.

The outcome probabilities are conditional on individual effects ($\alpha_{i}$) and the estimated coefficients can vary across the categories of the dependent variables.

### 3.1 Time preference

(7) $$\begin{array}{cc} & \mathrm{Pr}(H_{\mathrm{it}})=0|X_{\mathrm{it}}\text{,}Q_{\mathrm{it}}\text{,}P_{\mathrm{it}}\text{,}\alpha_{i}=\Phi (-(\beta_{0}X_{\mathrm{it}}+\xi_{0}Q_{\mathrm{it}}+\psi_{1}P_{\mathrm{it}}+\alpha_{i})\\ & \mathrm{Pr}(H_{\mathrm{it}})=1|X_{\mathrm{it}}\text{,}Q_{\mathrm{it}}\text{,}P_{\mathrm{it}}\text{,}\alpha_{i}=\Phi (-(\beta_{1}X_{\mathrm{it}}+\xi_{1}Q_{\mathrm{it}}+\psi_{1}P_{\mathrm{it}}+\alpha_{i})-\Phi (-\beta_{0}X_{\mathrm{it}}-\xi_{0}Q_{\mathrm{it}}-\psi_{1}P_{\mathrm{it}}-\alpha_{i})\\ & \vdots \\ & \mathrm{Pr}(H_{\mathrm{it}})=5|X_{\mathrm{it}}\text{,}Q_{\mathrm{it}}\text{,}P_{\mathrm{it}}\text{,}\alpha_{i}=\Phi (-(\beta_{5}X_{\mathrm{it}}+\xi_{5}Q_{\mathrm{it}}+\psi_{5}P_{\mathrm{it}}+\alpha_{i})-\Phi (-\beta_{5}X_{\mathrm{it}}-\xi_{5}Q_{\mathrm{it}}-\psi_{5}P_{\mathrm{it}}-\alpha_{i}) \end{array}$$

### 3.2 Risk preference

(8) $$\begin{array}{cc} & \mathrm{Pr}(R_{\mathrm{it}})=0|X_{\mathrm{it}}\text{,}Q_{\mathrm{it}}\text{,}P_{\mathrm{it}}\text{,}\alpha_{i}=\Phi (-(\beta_{0}X_{\mathrm{it}}+\xi_{0}Q_{\mathrm{it}}+\psi_{1}P_{\mathrm{it}}+\alpha_{i})\\ & \mathrm{Pr}(R_{\mathrm{it}})=1|X_{\mathrm{it}}\text{,}Q_{\mathrm{it}}\text{,}P_{\mathrm{it}}\text{,}\alpha_{i}=\Phi (-(\beta_{1}X_{\mathrm{it}}+\xi_{1}Q_{\mathrm{it}}+\psi_{1}P_{\mathrm{it}}+\alpha_{i})-\Phi (-\beta_{0}X_{\mathrm{it}}-\xi_{0}Q_{\mathrm{it}}-\psi_{1}P_{\mathrm{it}}-\alpha_{i})\\ & \mathrm{Pr}(R_{\mathrm{it}})=2|X_{\mathrm{it}}\text{,}Q_{\mathrm{it}}\text{,}P_{\mathrm{it}}\text{,}\alpha_{i}=\Phi (-(\beta_{2}X_{\mathrm{it}}+\xi_{2}Q_{\mathrm{it}}+\psi_{2}P_{\mathrm{it}}+\alpha_{i})-\Phi (-\beta_{2}X_{\mathrm{it}}-\xi_{2}Q_{\mathrm{it}}-\psi_{2}P_{\mathrm{it}}-\alpha_{i}) \end{array}$$

For the individual effects, $\alpha_{i}$ a zero mean and a constant variance is assumed.

We use the autofit option in the user written Stata programme (Pfarr et al., 2011) to conduct an iterative process to find which independent variables should be constrained and which should be unconstrained to equal cut points. The model is re-estimated until only variables that violate the equal cut-point assumption are identified. As a check on the results a Wald test is estimated on the best fit model with constraints on the appropriate explanatory variables to test the null hypothesis that the equal cut-point assumption is not violated. All models were estimated separately by offspring gender.

## 4 Results

The raw correlation in maternal and offspring time preference is 0.12 (*p*-value 0.000) for males and 0.16 (*p*-value 0.000) for females. The raw correlation in paternal and offspring time preference is 0.09 (*p*-value 0.000) for males and 0.12 (*p*-value 0.000) for females. The raw correlation in maternal and offspring risk preference is 0.09 (*p*-value 0.003) for males and 0.14 (*p*-value 0.000) for females. The raw correlation in paternal and offspring risk preference is 0.10 (*p*-value 0.0006) for males and 0.09 (*p*-value 0.0014) for females. There is therefore evidence of significant correlation between parental and offspring time and risk preference measures. In the raw data we also explored the correlation in risk and time preferences between parents and offspring between period *t* and *t*−1. We find for the majority of the sample the correlation between parents and offspring preferences measures are constant over time. This is more pronounced for parents and their daughters and for the risk preference measure. If there is a change in parental preferences between period *t* and *t−*1, for both parents and offspring the majority of the change is in moving one category in either direction as can be seen for offspring in Tables 1A and 1B. For extreme changes in parental preferences (i.e. moving from being future orientated to completely present orientated and from being risk averse to risk seeking), offspring preferences remain fairly constant over the same period suggesting that in the short term offspring may not be influenced by the same factors that lead to parents changing their preferences. This will be explored further and more robustly in the econometric analysis.

For ease of exposition, the main tables and figures only show the parental time and risk preference variables. The control variables for the best fit models are shown in Appendices A–C. Excluding the health behaviour variables did not change our results so the full models with all the explanatory variables are shown in the results tables.

### 4.1 Time preference (planning horizon)

The estimated coefficients from our base model, the random effects ordered probit model for the time preference proxy (planning horizon) are reported in Table 4. Note that the coefficients only have a qualitative interpretation. The results show that there is a highly significant relationship between time preference (planning horizon) of mothers and offspring. The magnitude of the partial effects (cut points) of the time preference variable varies between the categories. Only cut point 4 and 5 are significant for males, whilst cut points 2–5 are significant for females when maternal time preference is included and 3–5 when paternal time preference is included. This suggests that at least some of the explanatory variables have a different impact on each of the time preference categories and a more flexible model specification is required. Rho is highly significant for both genders suggesting that there is a correlation in the error term over time which we are able to control for in the analysis.

The results of the best fit generalised ordered probit model for the time preference proxy (planning horizon) are shown in Tables 5 for mothers and offspring and Table 6 for father and offspring. A list of the variables that do have a different impact on each of the time preference categories and the test coefficients from the global Wald Tests are presented in the notes sections of Tables 5 and 6.

Marginal effects are reported in Tables 3 and 4 to give the results a quantitative interpretation. Marginal effects are estimated for each preference category where the base category is the parent reporting a planning horizon of the next few days. The marginal effects on the diagonal, shaded in grey, are of main interest. They indicate how much more likely the offspring is to report each planning horizon if the parent has the same planning horizon rather than the shortest planning horizon (next week). In the case of mothers, all marginal effects are statistically significant. The correlations are generally larger for the longer planning horizons. Compared to the base category of reporting the shortest planning horizon, the strongest correlation is reporting a planning horizon of two to four years (3% for mothers and sons and 4% for mothers and daughters). The results are more mixed for fathers. Not all marginal effects on the diagonal are statistically significant. For fathers and sons, the strongest correlation is for reporting a planning horizon of the next few months compared to the base category of the shortest planning horizon. If a father has a planning horizon of the next few months his son is 5% more likely to report this planning horizon The largest correlation overall is for fathers and daughters reporting a planning horizon of one year compared to the base category of the shortest planning horizon.

Looking across the time preference categories, the strongest correlation of reporting the same time preference category is for mothers and daughters followed by mothers and sons, then fathers and daughters, and finally fathers and sons compared to the base category of reporting the shortest planning horizon. The relationship between parental and offspring time preference is most robust in the case of females and mothers.

Marginal effects can also be compared across offspring preference categories by parental planning horizon. Taking the parental planning horizon of 10+ years, we can see that marginal effects are generally increasing across the offspring planning horizons. That is, if the parent reports a planning horizon of 10+ year the likelihood of offspring reporting a particular planning horizon increases as the horizon increases. This relationship is slightly stronger for the mother and daughter dyad than the mother and son dyad. In Table 4, there is a similar pattern for father and offspring but the relationship is not as clear cut as the maternal and offspring time preference correlations.

**Fig. 1:**
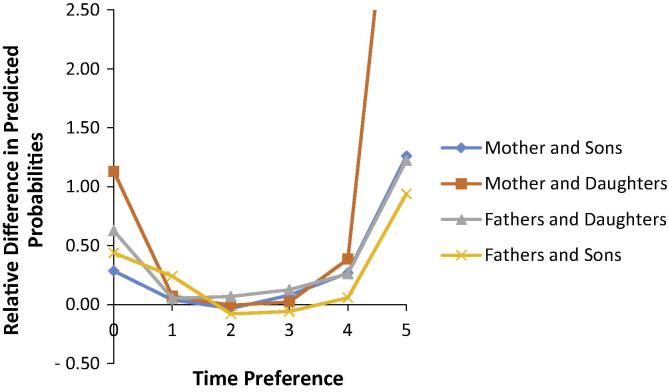
Relative difference in predicted probabilities of parents and offspring having same and conflicting time preference categories. Notes: On the *x*-axis, 0 = next week, 1 = next few months, 2 = next year, 3 = next 2–4 years, 4 = next 5–10 years, and 5 = 10+ years. The conflicting time preference category combinations are: offspring planning next week and parent planning 10+ years, offspring planning next few months and parent planning next 5–10 years, offspring planning the next year and parent planning next 2–4 years, offspring planning next 2–4 years and parent planning next year, offspring planning next 5–10 years and parent planning next few months, and finally, offspring planning next 10+ years and parent planning next week.

Additionally, to make the results easier to understand, we also estimate the relative difference between predicted probabilities for parents and offspring sharing the same time preference category and the predicted probabilities of parents and offspring having conflicting preference categories (for example, offspring having a planning horizon of next week and the parent having a planning horizon 10+ years, offspring having a planning horizon of next few months and the parent having a planning horizon of 5–10 years, etc.). If parents transmit their preferences to their offspring the predicted probabilities of sharing the same time preference category should be larger than the predicted probabilities of having conflicting time preference categories. The difference is expected to be largest at both ends of the planning horizon scale where the difference between the same and conflicting preferences is largest. Fig. 1 shows that the predicted probabilities for parents and offspring sharing the same preference category are always higher than parents and offspring holding conflicting time preference categories except for a few of the middle categories where the time length between the same and conflicting preference is smaller. As expected, the difference is higher at the extreme preference categories and lower for the middle preference categories where the time length between holding the same time planning horizon and conflicting planning horizons is not as extreme. The relative difference is larger for the longer planning horizon.

To summarise the results from the time preference models, overall, there is a larger association of maternal time preference on offspring time preference compared to the association with father’s time preference. The largest correlation is found for the longest planning horizons.

### 4.2 Risk preference (risk tolerance)

The base model for the risk preference proxy (risk tolerance) are reported in Table 7. Offspring are willing to accept more risk if their mothers are risk neutral rather than risk averse. The same holds if mothers are risk seeking but the effect is statistically significant in female offspring only. Both categories of father’s risk preference are significantly and positively associated with their son’s being willing to accept more risk. Having a father that is risk seeking is positive and significantly associated with their daughter being willing to accept more risk.

The magnitude of the cut-points varies by parent. Both risk preference categories are significant for offspring when maternal risk preference is included but only cut point 2 is statistically significant when paternal risk preference is included. This suggests that some of the explanatory variables have a different impact on each of the risk preference categories and a more flexible model specification is required. Rho is highly significant suggesting that the error term is correlated over time which is controlled for in the analysis.

Table 8 shows the marginal effects of the best fit generalised ordered probit models for risk preference. The variables that are permitted to vary between the risk preference categories and the global Wald Test for model fit are shown in the notes section of the table. The parallel cut-point assumption is not violated for maternal risk preference for males, in other words maternal risk preference has a homogenous effect on each of her son’s risk preference categories. Additionally, father’s risk preference did not violate the parallel line assumption in the father and son equations so that father’s risk preference has a homogenous effect on each of his son’s preference categories. Thus, overall for sons, parental risk preference has an equal effect on the son being either risk averse, risk-seeking, or risk neutral.

The marginal effects on the diagonal (shaded in grey) show that in the case of the mother daughter dyad and the father son dyad the correlation of both reporting risk neutral is higher compared to both reporting risk seeking. Compared to the base category of risk aversion, if a mother is risk neutral her daughter is 13% more likely to be risk neutral. This is the largest correlation overall. The marginal effects on the diagonal are not significant for the father daughter dyad. Overall, the stronger correlations in risk preferences are found between mothers and their offspring, followed by fathers and sons and then fathers and daughters.

When comparing marginal effects across offspring risk preference categories, the expected pattern is found in case of risk neutral parents. That is, if parents are risk neutral the marginal effects are larger for risk neutral offspring compared to risk seeking offspring. However in the case of risk seeking parents the marginal effects are larger for risk neutral offspring compared to risk seeking offspring in all dyads. For example, if a mother is risk seeking her son is 6% more likely to be risk neutral and 5% more likely to be risk seeking compared to the base category of risk aversion.

**Fig. 2:**
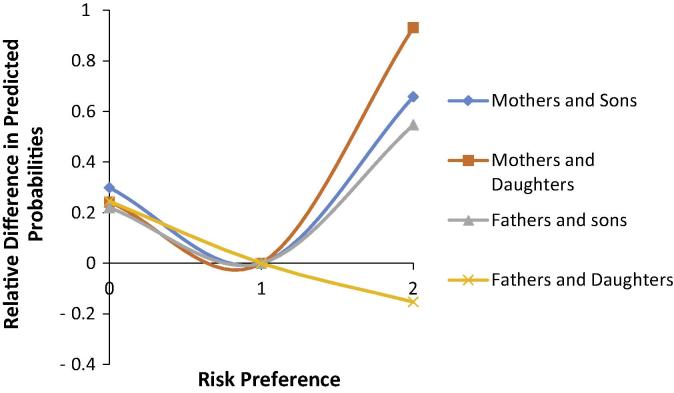
Relative difference in predicted probabilities of parents and offspring having same and conflicting risk preference categories. Notes: on the *x*-axis 0 = risk neutral, 1 = risk neutral, 2 = risk seeking. The conflicting risk preference categories are offspring risk averse and parent risk seeking, offspring risk neutral and parent risk neutral amd offspring risk seeking and parent risk averse.

Fig. 2 shows the relative difference in the predicted probabilities of parents sharing the same risk preference category compared to parents and offspring having conflicting risk preference categories (i.e. offspring risk averse and parent risk seeking, offspring risk neutral and parent risk neutral and offspring risk seeking and parent risk averse). The predicted probabilities for parents and offspring both reporting risk aversion and risk seeking preferences are always higher than parents and offspring holding conflicting risk preference categories except for father and daughter both reporting risk seeking. The relative difference is higher for sharing risk seeking preferences than risk averse preferences.

To summarise the results from the risk preference models, risk averse parents are more likely to have risk averse offspring, risk neutral parents are more likely to have risk neutral offspring, and risk seeking parents are more likely to have risk seeking offspring. The effect of parent risk preference is dependent on offspring gender. Daughters are more likely to be influenced by their mother’s risk preferences, however, sons are equally influenced by both parents.

## 5 Discussion and conclusion

This paper examined the correlation in offspring and parental time and risk preferences in new data (HILDA survey). The correlation in preferences between parents and their offspring was examined across the preference distribution and across the four parent–child dyads (mother/daughter, mother/son, father/daughter, father/son). The results show that there is a significant relationship between parents and their young adult offspring risk and time preference proxies (planning horizon and risk tolerance). Some gender differences were found. Research suggests that paternal involvement in intact families is dependent upon child gender, age, and race but is lower than maternal involvement (Harris, Furstenberg, & Marmer, 1998; Harris & Morgan, 1991). Mothers as the primary caregiver are likely to play a greater role in the formation of their offspring’s preferences. This is evident in our findings that the association in parental and offspring time preference was larger for mothers than fathers. Daughters were also more likely to be influenced by their mother’s risk preference behaviour. This may reflect that the extent of interaction and the intensity of the parental/offspring relationship is strongest across the same gender lines (Steinberg, 1987).

The results also showed that the correlation between parent and offspring time preference is not the same across the different preference categories. The largest correlation was found for the longest planning horizons suggesting that the transmission of patience (low rates of time preference) may be larger than transmission of impatience (high rates of time preference). This has not been previously investigated in the literature. Understanding the nuances of planning horizon is necessary for shedding light on how the mechanisms of the intergenerational transmission in time preference operates.

Our time preference results are largely consistent with other studies that look at both parents such as Webley and Nyhus (2006) although they did not separate by offspring gender. The correlation we find is generally smaller ranging between 2 percentage points to 6 percentage points compared to 5.2 percentage points in Kosse and Pfeiffer (2012) to 0.31 in Webley and Nyhus (2006). The magnitude of the correlation in parental and offspring risk preference is roughly consistent with the other studies such as Charles and Hurst (2003) and Hryshko et al. (2011). However, the use of different measures of time and risk preference and the use of different estimation techniques limits the comparability of results.

Our study is only the second study to examine both time and risk preferences. Our results are not directly comparable to Arrondel (2013) the only existing study we know of that looks at the intergenerational correlation in time and risk preference. In his analysis he does not estimate separate equations by gender and uses different proxies for time and risk preference. We find that the correlation varies across preferences (time and risk) and gender. This suggest the intergenerational relationship may be more nuanced than that observed by Aroundel.

There are several limitations to the study. Only proxies were available: planning horizon for time preference and willingness to take financial risk for risk preferences. As different proxies are used in the literature it makes it difficult to directly compare results. Parents and their offspring are observed at different points in the lifecycle. Whilst standard economic theory assumes that preferences are stable, preferences may vary over the lifecycle (Becker & Mulligan, 1997). The analysis controlled for age but a lifecycle bias may still have been resent. The age range of the offspring in our sample was 16–25, an important age range for making education and employment decisions. However, it may be the case that the correlation in preferences is stronger in this age group compared to older ages as parents may have a larger influence in younger offspring. Examining an older age group could provide insights into the persistence in the correlation in preferences when offspring leave their parental homes, start their own families and are more established in their employment and careers, etc. Finally, the majority of our sample (95%) resided with their parent(s) as either dependent students or non-dependent children. This is not representative of, and therefore results cannot necessarily be generalised to the whole population. In 2006 for example, approximately 42.7% of individuals in the 20–24 age group lived with their parents (ABS, 2009). It is likely that the influence of parent’s preferences on offspring preferences is larger when offspring are living at home. The correlation in preferences may therefore be smaller in more nationally representative samples.

One potential avenue for future research is further exploring the importance of parental gender in forming preferences. There are a number of pathways which may explain this gendered effect of correlation in risk preference. Parents are role models and children may learn about appropriate behaviour for their gender from observing their parent of the same gender and develop similar attitudes and behaviours. Or there may be other unobserved endowment effects as described in Becker and Tomes (1976) which may explain this association. Another avenue for future research is exploring if the transmission and formation of preferences differs between intact and single parent households. Research on the impact of non-resident fathers on children’s outcomes is mixed (Harris et al., 1998). Finally, examining the transmission of preferences across different age groups would be of interest. The influence of parents on their offspring’s time and risk preferences may vary across the lifecycle of their offspring. Understanding the formation and transmission of preferences may help to break the cycle of poverty as well as reduce health and income inequalities. The formation and transmission of preferences could be an integral part in the development of policy and interventions to improve educational attainment, health, and reduce inequalities.

## Figures and Tables

**Table:** 

time_preference	Next few months		Next year		Next 2–4 yrs		Next 5–10 yrs		Next 10+ yrs	
	ME	Std. Err.	ME	Std. Err.	ME	Std. Err.	ME	Std. Err.	ME	Std. Err.
*Males*										
age	0.01^∗∗^	(0.00)	0.01^∗∗^	(0.00)	0.00	(0.00)	0.00^∗^	(0.00)	−0.01^∗∗∗^	(0.00)
employed	0.02	(0.02)	−0.01	(0.02)	0.02	(0.02)	0.00	(0.01)	−0.01	(0.01)
unemployed	0.00	(0.00)	0.00	(0.01)	0.00	(0.01)	0.00	(0.01)	0.00	(0.01)
ft_student	0.03	(0.02)	0.03^∗^	(0.02)	0.00	(0.02)	0.00	(0.01)	−0.02^∗∗^	(0.01)
low income area	0.00	(0.00)	−0.01	(0.00)	−0.01	(0.01)	−0.01	(0.01)	−0.01	(0.01)
australian	0.00	(0.01)	0.00	(0.01)	−0.01	(0.02)	−0.01	(0.01)	−0.01	(0.01)
loghhincome	0.00	(0.00)	0.00	(0.00)	0.00	(0.01)	0.00	(0.00)	0.00	(0.00)
frequent physical activity	0.00^∗^	(0.00)	0.01^∗^	(0.00)	0.01^∗^	(0.01)	0.01^∗^	(0.00)	0.01^∗^	(0.00)
frequent drinker	0.00	(0.00)	0.00	(0.01)	0.00	(0.01)	0.00	(0.01)	0.00	(0.01)
smokes	0.01^∗∗^	(0.00)	−0.01^∗∗^	(0.00)	−0.02^∗∗^	(0.01)	−0.02^∗∗^	(0.01)	−0.02^∗∗^	(0.01)

*Mother variables*										
mother age	0.00	(0.00)	0.00	(0.00)	0.00	(0.00)	0.00	(0.00)	0.00	(0.00)
mother employed	0.00	(0.00)	0.00	(0.00)	0.00	(0.01)	0.00	(0.01)	0.00	(0.01)
mother unemployed	0.05	(0.08)	0.04	(0.07)	−0.01	(0.06)	−0.08^∗∗^	(0.04)	0.00	(0.04)
mother australian	0.00	(0.00)	−0.01	(0.00)	−0.01	(0.01)	−0.01	(0.01)	−0.01	(0.01)
mother highschool	0.01	(0.00)	−0.01	(0.01)	−0.02	(0.01)	−0.01	(0.01)	−0.01	(0.01)
mother some_higher_edu	0.00	(0.02)	−0.01	(0.02)	0.01	(0.02)	0.00	(0.01)	−0.01	(0.01)
mother degree	0.02	(0.02)	0.02	(0.02)	−0.01	(0.02)	0.01	(0.01)	−0.02^∗^	(0.01)
mother frequent physical activity	0.00	(0.00)	0.00	(0.00)	0.00	(0.01)	0.00	(0.00)	0.00	(0.00)
mother frequent drinker	−0.02	(0.02)	0.02	(0.02)	0.02	(0.02)	0.00	(0.01)	0.01	(0.01)
mother smoker	−0.06^∗∗^	(0.02)	0.01	(0.02)	−0.01	(0.02)	−0.02	(0.01)	−0.01	(0.01)

*Females*										
age	0.01^∗∗^	(0.00)	0.00	(0.00)	−0.01^∗^	(0.00)	0.00	(0.00)	0.00^∗^	(0.00)
employed	0.01	(0.02)	0.04^∗∗^	(0.02)	0.00	(0.01)	−0.01	(0.01)	−0.01^∗∗^	(0.01)
unemployed	0.02^∗∗^	(0.01)	−0.01^∗∗^	(0.01)	−0.03^∗∗^	(0.01)	−0.02^∗∗^	(0.01)	−0.01^∗∗^	(0.01)
ft_student	0.07^∗∗∗^	(0.02)	−0.02	(0.02)	0.01	(0.02)	0.02	(0.01)	−0.01	(0.01)
two_parent hh	0.04^∗∗^	(0.02)	−0.02	(0.02)	0.00	(0.02)	0.02^∗^	(0.01)	−0.02^∗∗^	(0.01)
low income area	0.00	(0.01)	0.00	(0.00)	0.01	(0.01)	0.00	(0.01)	0.00	(0.00)
australian	0.00	(0.01)	0.00	(0.01)	0.00	(0.01)	0.00	(0.01)	0.00	(0.01)
loghhincome	−0.01	(0.00)	0.00	(0.00)	0.01	(0.01)	0.01	(0.00)	0.00	(0.00)
frequent physical activity	−0.01^∗∗^	(0.00)	0.01	(0.00)	0.02^∗∗^	(0.01)	0.01^∗∗^	(0.00)	0.01^∗∗^	(0.00)
frequent drinker	0.02	(0.01)	−0.01	(0.01)	−0.03	(0.02)	−0.02	(0.01)	−0.01	(0.01)
smokes	0.02^∗∗∗^	(0.01)	−0.02^∗∗∗^	(0.00)	−0.04^∗∗∗^	(0.01)	−0.02^∗∗^	(0.01)	−0.02^∗∗∗^	(0.00)

*Mother variables*										
mother age	0.00^∗∗^	(0.00)	0.00^∗∗^	(0.00)	0.00^∗∗^	(0.00)	0.00^∗∗^	(0.00)	0.00^∗∗^	(0.00)
mother employed	0.00	(0.01)	0.00	(0.00)	0.00	(0.01)	0.00	(0.01)	0.00	(0.00)
mother unemployed	0.01	(0.05)	−0.04	(0.04)	0.03	(0.04)	−0.02	(0.02)	0.04^∗∗^	(0.02)
mother australian	0.00	(0.01)	0.00	(0.00)	0.00	(0.01)	0.00	(0.01)	0.00	(0.00)
mother highschool	0.00	(0.01)	0.00	(0.01)	0.00	(0.01)	0.00	(0.01)	0.00	(0.00)
mother some_higher_edu	0.01	(0.02)	0.01	(0.02)	−0.01	(0.02)	0.01	(0.01)	−0.03^∗∗∗^	(0.01)
mother degree	0.05^∗∗^	(0.02)	0.02	(0.02)	−0.02	(0.02)	−0.01	(0.01)	−0.02^∗∗^	(0.01)
mother frequent physical activity	0.01^∗∗^	(0.00)	−0.01^∗∗^	(0.00)	−0.01^∗∗^	(0.01)	−0.01^∗∗^	(0.00)	−0.01^∗∗^	(0.00)
mother frequent drinker	0.04^∗^	(0.02)	0.03	(0.02)	−0.02	(0.02)	−0.03^∗∗^	(0.01)	−0.01	(0.01)
mother smoker	−0.01	(0.02)	0.00	(0.02)	−0.05^∗∗^	(0.02)	0.03^∗∗^	(0.01)	0.00	(0.01)

**Table:** 

time_preference	Next few months		Next year		Next 2–4 yrs		Next 5–10 yrs		Next 10+ yrs	
	ME	Std. Err.	ME	Std. Err.	ME	Std. Err.	ME	Std. Err.	ME	Std. Err.
*Males*										
age	0.01^∗∗∗^	(0.00)	0.01^∗∗^	(0.00)	0.00	(0.00)	0.00^∗^	(0.00)	−0.01^∗∗∗^	(0.00)
employed	0.00	(0.00)	0.00	(0.00)	0.00	(0.01)	0.00	(0.01)	0.00	(0.01)
unemployed	−0.07^∗∗^	(0.03)	0.02	(0.03)	0.02	(0.02)	−0.03^∗^	(0.02)	0.01	(0.01)
ft_student	0.00	(0.00)	0.00	(0.00)	0.00	(0.01)	0.00	(0.01)	0.00	(0.01)
low income area	0.00	(0.00)	0.00	(0.00)	−0.01	(0.01)	−0.01	(0.01)	−0.01	(0.01)
australian	0.01	(0.01)	−0.01	(0.01)	−0.01	(0.01)	−0.01	(0.01)	−0.01	(0.01)
loghhincome	0.00	(0.00)	0.00	(0.00)	0.00	(0.01)	0.00	(0.00)	0.00	(0.00)
frequent physical activity	0.00^∗^	(0.00)	0.01^∗^	(0.00)	0.01^∗^	(0.01)	0.01^∗^	(0.00)	0.01^∗^	(0.00)
frequent drinker	−0.03	(0.03)	0.01	(0.03)	−0.05^∗∗^	(0.02)	0.00	(0.02)	0.03^∗∗^	(0.01)
smokes	−0.05^∗∗^	(0.02)	−0.03	(0.02)	−0.04^∗∗^	(0.02)	0.00	(0.01)	0.01	(0.01)

*Father variables*										
father age	0.00^∗^	(0.00)	0.00^∗^	(0.00)	0.00^∗^	(0.00)	0.00^∗^	(0.00)	0.00^∗^	(0.00)
father employed	−0.01^∗^	(0.00)	0.01^∗^	(0.01)	0.02^∗^	(0.01)	0.02^∗^	(0.01)	0.02^∗^	(0.01)
father unemployed	−0.02	(0.01)	0.02	(0.01)	0.04	(0.02)	0.03	(0.02)	0.03	(0.02)
father australian	0.00	(0.00)	0.00	(0.00)	0.01	(0.01)	0.01	(0.01)	0.01	(0.01)
father highschool	0.01	(0.01)	−0.01	(0.01)	−0.02	(0.01)	−0.01	(0.01)	−0.01	(0.01)
father some_higher_edu	0.00	(0.00)	0.00	(0.00)	−0.01	(0.01)	−0.01	(0.01)	−0.01	(0.01)
father degree	0.07^∗∗^	(0.02)	0.00	(0.02)	0.02	(0.02)	0.01	(0.01)	−0.02	(0.01)
father frequent physical activity	0.01^∗∗^	(0.00)	−0.01^∗∗^	(0.00)	−0.01^∗∗^	(0.01)	−0.01^∗∗^	(0.00)	−0.01^∗∗^	(0.00)
father frequent drinker	0.00	(0.00)	0.00	(0.00)	0.00	(0.01)	0.00	(0.00)	0.00	(0.00)
father smoker	0.00	(0.00)	−0.01	(0.00)	−0.01	(0.01)	−0.01	(0.01)	−0.01	(0.01)

*Females*										
age	0.01	(0.00)	0.01^∗∗^	(0.00)	−0.01^∗^	(0.00)	0.00	(0.00)	0.00^∗^	(0.00)
employed	0.00	(0.01)	0.00	(0.00)	0.00	(0.01)	0.00	(0.01)	0.00	(0.00)
unemployed	0.01	(0.01)	−0.01	(0.01)	−0.02	(0.01)	−0.01	(0.01)	−0.01	(0.01)
ft_student	0.07^∗∗^	(0.02)	−0.02	(0.02)	0.02	(0.02)	0.00	(0.01)	−0.01	(0.01)
low income area	−0.01	(0.01)	0.00	(0.00)	0.01	(0.01)	0.01	(0.01)	0.00	(0.00)
australian	0.00	(0.01)	0.00	(0.01)	0.00	(0.02)	0.00	(0.01)	0.00	(0.01)
loghhincome	0.00	(0.01)	0.00	(0.00)	0.00	(0.01)	0.00	(0.01)	0.00	(0.00)
frequent physical activity	−0.01^∗∗^	(0.00)	0.01^∗∗^	(0.00)	0.02^∗∗^	(0.01)	0.01^∗∗^	(0.00)	0.01^∗∗^	(0.00)
frequent drinker	0.02	(0.01)	−0.01	(0.01)	−0.02	(0.02)	−0.02	(0.01)	−0.01	(0.01)
smokes	0.02^∗∗^	(0.01)	−0.02^∗∗^	(0.01)	−0.03^∗∗^	(0.01)	−0.02^∗∗^	(0.01)	−0.01^∗∗^	(0.01)

*Father variables*										
father age	0.00^∗∗^	(0.00)	0.00^∗∗^	(0.00)	0.00^∗∗^	(0.00)	0.00^∗∗^	(0.00)	0.00^∗∗^	(0.00)
father employed	−0.01	(0.01)	0.01	(0.01)	0.02	(0.01)	0.01	(0.01)	0.01	(0.01)
father unemployed	0.03	(0.02)	−0.02	(0.01)	−0.04	(0.03)	−0.03	(0.02)	−0.02	(0.01)
father australian	0.00	(0.01)	0.00	(0.00)	0.01	(0.01)	0.00	(0.01)	0.00	(0.00)
father highschool	−0.02	(0.01)	0.01	(0.01)	0.02	(0.02)	0.02	(0.01)	0.01	(0.01)
father some_higher_edu	0.00	(0.01)	0.00	(0.00)	0.00	(0.01)	0.00	(0.01)	0.00	(0.00)
father degree	0.06^∗∗^	(0.02)	0.02	(0.02)	−0.02	(0.02)	0.00	(0.01)	−0.02^∗∗^	(0.01)
father frequent physical activity	0.02^∗∗^	(0.00)	−0.01^∗∗∗^	(0.00)	−0.02^∗∗∗^	(0.01)	−0.02^∗∗∗^	(0.00)	−0.01^∗∗^	(0.00)
father frequent drinker	0.01^∗∗∗^	(0.01)	−0.01^∗∗^	(0.00)	−0.02^∗∗^	(0.01)	−0.01^∗∗^	(0.01)	−0.01^∗∗^	(0.00)
father smoker	0.00	(0.01)	0.00	(0.00)	−0.01	(0.01)	0.00	(0.01)	0.00	(0.00)

**Table:** 

risk_preference	Males				Females			
	Risk neutral		Risk seeking		Risk neutral		Risk seeking	
	ME	Std. Err.	ME	Std. Err.	ME	Std. Err.	ME	Std. Err.
*Mothers*								
age	0.02^∗∗∗^	(0.01)	0.00	(0.00)	0.00	(0.00)	0.00	(0.00)
employed	−0.02	(0.02)	−0.02	(0.01)	−0.01	(0.02)	0.00	(0.01)
unemployed	0.00	(0.03)	0.00	(0.02)	0.02	(0.04)	0.01	(0.02)
ft_student	0.00	(0.02)	0.00	(0.01)	0.02	(0.02)	0.01	(0.01)
two_parent hh	−0.01	(0.02)	−0.01	(0.02)	−0.03	(0.03)	−0.01	(0.01)
low income area	−0.05^∗∗^	(0.02)	−0.04^∗∗^	(0.02)	−0.01	(0.03)	0.00	(0.01)
australian	−0.03	(0.03)	−0.02	(0.03)	−0.05	(0.04)	−0.02	(0.01)
loghhincome	0.02	(0.01)	0.01	(0.01)	0.02	(0.02)	0.01	(0.01)
frequent physical activity	−0.02	(0.01)	−0.02	(0.01)	0.01	(0.02)	0.01	(0.01)
frequent drinker	0.06^∗∗^	(0.02)	0.05^∗∗^	(0.02)	0.05	(0.05)	0.02	(0.02)
smokes	−0.06^∗^	(0.03)	0.04^∗^	(0.02)	0.06^∗^	(0.03)	0.02^∗^	(0.01)

*Mother variables*								
mother age	0.00	(0.00)	0.00	(0.00)	0.00	(0.00)	0.00	(0.00)
mother employed	0.01	(0.02)	0.01	(0.02)	0.02	(0.03)	0.01	(0.01)
mother unemployed	−0.06	(0.07)	−0.05	(0.06)	−0.02	(0.08)	−0.01	(0.03)
mother australian	0.01	(0.02)	0.01	(0.02)	−0.03	(0.02)	−0.01	(0.01)
mother highschool	−0.04^∗^	(0.02)	−0.04^∗^	(0.02)	−0.03	(0.03)	−0.01	(0.01)
mother some_higher_edu	−0.04^∗^	(0.02)	−0.03^∗^	(0.02)	0.01	(0.03)	0.00	(0.01)
mother degree	−0.04^∗^	(0.02)	−0.03^∗^	(0.02)	0.08^∗∗^	(0.03)	−0.03^∗^	(0.01)
mother frequent physical activity	0.00	(0.01)	0.00	(0.01)	−0.01	(0.02)	0.00	(0.01)
mother frequent drinker	−0.01	(0.02)	−0.01	(0.01)	0.01	(0.02)	0.00	(0.01)
mother smoker	−0.08^∗∗^	(0.03)	0.04^∗^	(0.02)	−0.01	(0.03)	−0.01	(0.01)

*Fathers*								
age	0.02^∗∗^	(0.01)	0.00	(0.00)	0.00	(0.01)	0.00	(0.00)
employed	−0.03	(0.02)	−0.03^∗^	(0.01)	−0.03	(0.03)	−0.01	(0.01)
unemployed	−0.01	(0.03)	−0.01	(0.03)	0.02	(0.05)	0.00	(0.02)
ft_student	0.00	(0.02)	0.00	(0.01)	0.00	(0.03)	0.00	(0.01)
low income area	−0.03	(0.02)	−0.03	(0.02)	−0.10^∗∗^	(0.04)	0.02	(0.02)
australian	0.00	(0.04)	0.00	(0.03)	−0.08^∗^	(0.05)	−0.03^∗^	(0.01)
loghhincome	0.00	(0.01)	0.00	(0.01)	0.01	(0.02)	0.00	(0.01)
frequent physical activity	−0.04^∗∗^	(0.02)	−0.03^∗∗^	(0.01)	0.01	(0.02)	0.00	(0.01)
frequent drinker	0.11^∗∗∗^	(0.03)	0.09^∗∗∗^	(0.02)	0.01	(0.06)	0.00	(0.02)
smokes	−0.07^∗^	(0.04)	0.04^∗^	(0.02)	0.06	(0.04)	0.02	(0.01)

*Father variables*								
father age	0.00	(0.00)	0.00	(0.00)	0.00	(0.00)	0.00	(0.00)
father employed	0.00	(0.03)	0.00	(0.03)	0.02	(0.05)	0.01	(0.02)
father unemployed	0.04	(0.07)	0.04	(0.06)	−0.05	(0.09)	−0.01	(0.03)
father australian	0.01	(0.02)	0.01	(0.02)	0.00	(0.03)	0.00	(0.01)
father highschool	0.09^∗∗^	(0.03)	0.08^∗∗^	(0.03)	−0.06	(0.06)	−0.02	(0.02)
father some_higher_edu	−0.05^∗^	(0.03)	0.04^∗^	(0.02)	−0.02	(0.04)	−0.01	(0.01)
father degree	0.01	(0.03)	0.01	(0.02)	0.05	(0.04)	0.01	(0.01)
father frequent physical activity	0.00	(0.01)	0.00	(0.01)	0.01	(0.02)	0.00	(0.01)
father frequent drinker	−0.02	(0.02)	−0.02	(0.01)	0.03	(0.02)	0.01	(0.01)
father smoker	−0.11^∗∗∗^	(0.03)	0.05^∗∗^	(0.02)	0.04	(0.03)	0.01	(0.01)

**Table 1A:** Time preference 16–25 year olds.

Notes: Columns are time preference in period *t*−1, rows are time preference in period *t.*

**Table 1B:** Risk preference 16–25 years olds.

Notes: Columns are risk preference in period *t−*1, row are risk preference in period *t.*

**Table 2:** Variable list and description.

Variable	Description	HILDA code
*Dependent variable*		
Time preference	0-next week	fisavep
1-next few months	
2-next year	
3-next 2–4 years	
4-next 5–10 year	
5-more than 10 years	
Risk preference	0-no risk	firisk
1-risk neutral	
2-risk seeking	

*Individual characteristics*		
Age	Age in years	hgage
Australian	0 = Not born in Australia	ancob
1 = Born in Australia	
Employed	0 = Unemployed/Not in the labour force	esbrd
1 = Employed	
Unemployed	0 = Employed/Not in the labour force	esbrd
1 = Employed	
Ft student	0-not currently a full time student	
	1-currently a full time student	edfts
Frequent drinker	0-drinks alcohol less than 5 times per week	lsdrkf
1-drinks alcohol more than 5 times per week	
Frequent exercise	0-participates in physical activity < 3× per week	lspact
1-participates in physical activity < 3× per week	
Smoker	0-not current smoker	lssmkf
1-current smoker	

*Household characteristics*		
Loghhincome	Log(Household income/household size)	Hifefp/hhpers
Low income area^∗^	0 = Index of socioeconomic disadvantage four least deprived deciles (7–10)	hhda10
1 = Lives in an area classified by the index of socioeconomic disadvantage as the 3 most deprived deciles (1–3)	
Two parent hh	0-only mother in household	Derived from hhfxid
1-both mother and father live together	

*Parental characteristics*		
High school	0 = No qualifications	edhigh
1 = High school degree equivalent (year 12 education)	
Some higher edu.	0 = No qualifications	edhigh
1 = Certificates I–IV, Diploma, Advanced Diploma	
Degree	0 = No qualifications	edhigh
1 = First degree or higher	
Age	Age in years	hgage
Employed	0 = Unemployed/Not in the labour force	esbrd
1 = Employed	
Unemployed	0 = Employed/Not in the labour force	esbrd
1 = Employed	
Frequent drinker	0-drinks alcohol less than 5 times per week	lsdrkf
1-drinks alcohol more than 5 times per week	
Frequent exercise	0-participates in physical activity < 3× per week	lspact
1-participates in physical activity < 3× per week	
Smoker	0-not current smoker	lssmkf
1-current smoker	
Time preference	0-next week	fisavep
1-next few months	
2-next year	
3-next 2–4 years	
4-next 5–10 year	
5-more than 10 years	
Risk preference	0-no risk	firisk
1-risk neutral	
2-risk seeking	

Notes: ^∗^Index of socio-economic disadvantage is calculated using areas defined from the 2001 Australian census and is based on attributes such as low income, low educational attainment, and high unemployment. There are ten deciles with higher numbers indicating lower levels of deprivation.

**Table 3:** Descriptive statistics.

	Males			Females			Mothers			Fathers		
	*n*	%		*n*	%		*n*	%		*n*	%	
*Planning horizon*												
next week	1374	0.32		1026	0.25		1021	0.24		525	0.18	
next few months	1290	0.30		1199	0.29		1103	0.26		768	0.27	
next year	661	0.15		818	0.20		671	0.16		506	0.18	
2–4 years	565	0.13		632	0.15		450	0.11		344	0.12	
5–10 years	284	0.07		279	0.07		576	0.14		517	0.18	
>10 years	186	0.04		132	0.03		364	0.09		226	0.08	

*Risk tolerance*												
risk averse	1034	0.49		1215	0.61		2007	0,54		520	0.36	
risk neutral	676	0.40		676	0.34		1458	0.39		727	0.51	
risk seeking	228	0.11		94	0.05		247	0.07		188	0.13	

	Mean	St dev	*n*	Mean	St dev	*n*	Mean	St dev	*n*	Mean	St dev	*n*
age	18.54	(2.82)	9430	18.26	(2.75)	8435	46.71	(6.58)	7811	49.17	7.33	5746
employed	0.61	(0.49)	8411	0.63	(0.48)	7658	0.72	(0.45)	7424	0. 87	(0.33)	5084
unemployed	0.10	(0.30)	8411	0.08	(0.27)	7658	0.03	(0.17)	7424	0.02	(0.14)	5084
ft student	0.53	(0.50)	8411	0.61	(0.49)	7658	–	–	–	–	–	–
2 parent household	0.78	(0.41)	9385	0.78	(0.41)	8396	–	–	–	–	–	–
loghhincome	9.99	(0.65)	9398	10.02	(0.64)	4363	10.01	(0.68)	7781	10.08	(0.67)	5674
low income area	0.28	(0.45)	9430	0.25	(0.43)	8435	0.29	(0.45)	7811	0.26	(0.44)	5698
Australian	0.91	(0.28)	8408	0.91	(0.29)	7658	0.72	(0.44)	7424	0.71	(0.45)	5079
frequent physical activity	0.68	(0.47)	7249	0.50	(0.50)	6876	0.44	(0.50)	6713	0.50	(0.50)	4594
frequent drinker	0.08	(0.28)	7410	0.03	(0.17)	7023	0.22	(0. 41)	6776	0.40	(0.49)	4686
smoker	0.19	(0.39)	6537	0.13	(0.34)	6243	0. 22	(0.42)	6077	0.22	(0.42)	4162
highschool	–	–	–	–	–	–	0.29	(0.45)	7424	0.08	(0.27)	5084
some higher education	–	–	–	–	–	–	0.22	(0.42)	7424	0.44	(0.50)	5084
degree	–	–	–	–	–	–	0.44	(0.50)	7424	0.24	(0.43)	5084

Notes: Loghhincome is log of household income measured in Australian dollars. Age is measured in years. All other variables are percentages. The parent sample is adjusted for families with multiple children in the sample. Only one observation for each year for each parent is included in the table above.

**Table 4:** Random effects ordered probit for time preference (planning horizon) mothers and offspring.

Planning horizon	Mothers				Fathers			
	Males		Females		Males		Females	
	Coef.	Std. Err.	Coef.	Std. Err.	Coef.	Std. Err.	Coef.	Std. Err.
*Parent planning horizon*								
plan next few months	0.15^∗∗^	(0.06)	0.21^∗∗^	(0.07)	0.14^∗^	(0.08)	0.15^∗^	(0.08)
plan next year	0.24^∗∗^	(0.07)	0.43^∗∗∗^	(0.08)	0.29^∗∗^	(0.09)	0.25^∗∗^	(0.09)
plan next 2–4 years	0.34^∗∗∗^	(0.08)	0.46^∗∗∗^	(0.09)	0.20^∗∗^	(0.10)	0.35^∗∗^	(0.10)
plan next 5–10 years	0.33^∗∗∗^	(0.08)	0.45^∗∗∗^	(0.08)	0.30^∗∗^	(0.09)	0.34^∗∗∗^	(0.10)
plan 10 years+	0.34^∗∗∗^	(0.09)	0.61^∗∗∗^	(0.10)	0.38^∗∗∗^	(0.11)	0.44^∗∗∗^	(0.12)
cut 1	−0.69	(0.45)	0.74	(0.48)	−0.74	(0.53)	−0.17	(0.58)
cut 2	0.22	(0.45)	1.74^∗∗∗^	(0.48)	0.21	(0.53)	0.87	(0.58)
cut 3	0.74	(0.45)	2.41^∗∗∗^	(0.49)	0.71	(0.53)	1.53^∗∗^	(0.58)
cut 4	1.38^∗∗^	(0.45)	3.21^∗∗∗^	(0.49)	1.36^∗∗^	(0.53)	2.32^∗∗∗^	(0.58)
cut 5	1.94^∗∗∗^	(0.45)	3.91^∗∗∗^	(0.49)	1.93^∗∗∗^	(0.53)	3.04^∗∗∗^	(0.58)
rho	0.27^∗∗∗^	(0.02)	0.30^∗∗∗^	(0.03)	0.28^∗∗∗^	(0.03)	0.29^∗∗∗^	(0.03)
log likelihood	−5230.14		−4858.78		−3971.75		−3624.90	
observations	3364		3131		2527		2326	

Notes: Standard errors are in parenthesis. ^∗∗∗^ indicates significance at *p* < 0.001, ^∗∗^ indicates significance at the *p* < 0.05, and ^∗^ indicates significance at *p* < 0.10.

**Table 5:** Generalised random effects ordered probit model offspring and mothers (best fit model).

Notes: Standard errors are in parenthesis. ^∗∗∗^ indicates significance at *p* < 0.001, ^∗∗^ indicates significance at the *p* < 0.05, and ^∗^ indicates significance at *p* < 0.10. Marginal effects are shown and are calculated for the probability of a positive outcome in each time preference category. Males: two_parent hh dropped from the model because of multicollinearity. The variables that are not constrained to parallel cut-points are age, frequent drinker, smokes, mother plans year, and mother age. The global Wald test to test the hypothesis that parallel line assumption is not violated in the best fit model: *χ*^2^ = 97.10 and *p* = 0.1555 implying that this is the best fit model. Females: the variables that are not constrained to parallel cut-points are age, employed, ft time student, two_parent_hh mother plans 10+ years, mother unemployed, mother some_higher_edu, mother degree, mother frequent drinker, and mother smokes. The global Wald test to test the hypothesis that parallel line assumption is not violated in the best fit model: *χ*^2^ = 64.65 and *p* = 0.4537 implying that this is the best fit model.

**Table 6:** Generalised random effects ordered probit model offspring and fathers (best fit model).

Notes: Standard errors are in parenthesis. ^∗∗∗^ indicates significance at *p* < 0.001, ^∗∗^ indicates significance at the *p* < 0.05, and ^∗^ indicates significance at *p* < 0.10. Marginal effects are shown and are calculated for the probability of a positive outcome in each time preference category. Males: the variables that are not constrained to parallel cut-points are age, unemployed, frequent drinker, smokes, father plan month, father plan 2–4 years, father degree. The global Wald test to test the hypothesis that parallel line assumption is not violated in the best fit model: *χ*^2^ = 81.01 and *p* = 0.2186 implying that this is the best fit model. Females: The variables that are not constrained to parallel cut-points are age, ft_student, father plan year, father plan 2–4 years, and father degree. The global Wald test to test the hypothesis that parallel line assumption is not violated in the best fit model: *χ*^2^ = 70.22 and *p* = 0.7744 implying that this is the best fit model.

**Table 7:** Random effects ordered probit for risk preference (risk tolerance) parents and offspring.

Risk tolerance	Mother				Father			
	Males		Females		Males		Females	
	Coef.	Std. Err.	Coef.	Std. Err.	Coef.	Std. Err.	Coef.	Std. Err.
*Parent risk tolerance*								
risk neutral	0.14^∗∗^	(0.08)	0.30^∗∗∗^	(0.09)	0.29^∗∗^	(0.10)	0.12	(0.12)
risk seeking	0.39	(0.16)	0.48^∗∗^	(0.18)	0.31^∗∗^	(0.15)	0.34^∗∗^	(0.16)
cut 1	2.06^∗∗^	(0.83)	1.58^∗^	(0.93)	0.95	(0.98)	0.99	(1.11)
cut 2	3.78^∗∗∗^	(0.84)	3.49^∗∗∗^	(0.93)	2.75^∗∗^	(0.99)	2.98^∗∗^	(1.11)
Rho	0.38^∗∗∗^	(0.05)	0.45^∗∗∗^	(0.05)	0.43^∗∗∗^	(0.05)	0.44^∗∗∗^	(0.06)
log likelihood	−1390.32		−1148.32		−1124.96		−909.86	
Observations	1539		1480					

Notes: Standard errors are in parenthesis. ^∗∗∗^ indicates significance at *p* < 0.001, ^∗∗^ indicates significance at the *p* < 0.05, and ^∗^ indicates significance at *p* < 0.10.

**Table 8:** Generalised random effects ordered probit for risk preference offspring and parents (best fit model).

Notes: Standard errors are in parenthesis. ^∗∗∗^ indicates significance at *p* < 0.001, ^∗∗^ indicates significance at the *p* < 0.05, and ^∗^ indicates significance at *p* < 0.10. Marginal effects are shown and are calculated for the probability of a positive outcome in each time preference category. For males, the variables that are not constrained to parallel cut-points are age, smoker and mother smoker. For females, the variables that are not constrained to parallel cut-points are mother risk neutral and mother degree. For males & mothers, the global Wald test to test the hypothesis that parallel line assumption is not violated in the best fit model: *χ*^2^ = 19.01 and *p* = 0.5209 implying that this is the best fit model. For females & mothers, the global Wald test to test the hypothesis that parallel line assumption is not violated in the best fit model: *χ*^2^ = 1 = 23.79 and *p* = 0.3035 implying that this is the best fit model. For males & fathers, the variables that are not constrained to parallel cut-points are age, smokes, father some higher education, and father smokes. For females & fathers, the variables that are not constrained to parallel cut-points are low income area and father risk seeking. For males, the global Wald test to test the hypothesis that parallel line assumption is not violated in the best fit model: *χ*^2^ = 15.28 and *p* = 0.6424 implying that this is the best fit model. For females, the global Wald test to test the hypothesis that parallel line assumption is not violated in the best fit model: *χ*^2^ = 1 = 18.28 and *p* = 0.5688 implying that this is the best fit model.

## Appendix A Generalised random effects ordered probit for time preference mothers and offspring (best fit)

Notes: Additional covariates from Table 4.

## Appendix B Generalised random effects ordered probit model for time preference fathers and offspring (best fit)

Notes: Additional covariates from Table 5.

## Appendix C Generalised random effects ordered model for risk preference offspring and parents (best fit model)

Notes: Additional covariates from Table 7.

## Footnotes

1 Wave 11 is only included in the risk preference models.

2 Wave 1 is not used as the smoking question and alcohol question changed from wave 1 to 2. Therefore to ensure comparability between waves we start with data from wave 2. Waves 5, 7, and 9 are not included because these waves do not ask respondents the questions used to proxy for time and risk preference.

3 The tables for parents are available upon request to the authors.
